# *bioSyntax*: syntax highlighting for computational biology

**DOI:** 10.1186/s12859-018-2315-y

**Published:** 2018-08-22

**Authors:** Artem Babaian, Anicet Ebou, Alyssa Fegen, Ho Yin Kam, German E. Novakovsky, Jasper Wong, Dylan Aïssi, Li Yao

**Affiliations:** 1 Terry Fox Laboratory, BC Cancer Research Centre, 675 West 10th Avenue, Vancouver, BC V5Z 1L3 Canada; 2 Department of Medical Genetics, University of British Columbia, Vancouver, BC Canada; 3 Departement de Formation et de Recherches Agriculture et Ressources Animales, Institut National Polytechnique Felix Houphouet-Boigny, Yamoussoukro, Côte d’Ivoire; 4 Faculty of Science, University of British Columbia, Vancouver, BC Canada; 5 Faculty of Mathematics, University of Waterloo, Waterloo, ON Canada; 6 Genome Science and Technology, University of British Columbia, Vancouver, BC Canada; 7 Department of General and Interventional Cardiology, University Heart Center Hamburg, Hamburg, Germany; 8 THU-PKU Joint Center for Life Sciences, Tsinghua University, Beijing, China

**Keywords:** Syntax highlighting, *Vim*, *Sublime*, Command line interface, SAM, VCF, FASTA, FASTQ

## Abstract

**Background:**

Computational biology requires the reading and comprehension of biological data files. Plain-text formats such as SAM, VCF, GTF, PDB and FASTA, often contain critical information which is obfuscated by the data structure complexity.

**Results:**

*bioSyntax* (https://biosyntax.org/) is a freely available suite of biological syntax highlighting packages for *vim, gedit*, *Sublime*, *VSCode,* and *less. bioSyntax* improves the legibility of low-level biological data in the bioinformatics workspace.

**Conclusion:**

*bioSyntax* supports computational scientists in parsing and comprehending their data efficiently and thus can accelerate research output.

**Electronic supplementary material:**

The online version of this article (10.1186/s12859-018-2315-y) contains supplementary material, which is available to authorized users.

## Background

A major component of computational biology research involves the reading and comprehension of data in biological file-formats including FASTA [1], FASTQ [2], gene transfer format (GTF) [3], variant calling format (VCF) [4], protein database format (PDB) [5, 6], and sequence alignment map (SAM) [7] amongst others [8]. While being easy to parse computationally, these and other biological files often become illegible as their size and complexity increases, including header sections that contain critical data descriptors often required for downstream processing.

Syntax highlighting is designed to improve the interpretability of text files with well-defined structures through the application of colour, font, and formatting to differentiate content; typically a set of keywords, structures, and symbols. Originally developed for the code editor Emily in 1971 [9] and later *LEXX* in the late 1980s [10], syntax highlighting is now ubiquitous in the computer sciences. There are contradictory findings whether syntax highlighting offers performance benefits on computer programming comprehension or efficiency but syntax highlighting consistently decreases the reported difficulty of work tasks [11–15]. In terms of human perception, colour (via syntax highlighting) increases the visual saliency of data thereby decreasing search times to find a particular symbol in a visual search task [16–18].

A plethora of tools, both stand-alone and web-based, exist to help scientists visualize and process biological data [19–25]. However, there is no comprehensive set of software to assist in direct inspection and interpretation of raw biological data and their headers.

The objective of *bioSyntax* is to improve the human readability of scientific data-formats through seamlessly integrated syntax highlighting and to assist scientists in performing visual search tasks when working with low-level data.

## Implementation

*bioSyntax* is currently ported for four common text editors, *Vim* (and *GVim*), *gedit* (and other linux editors using the *GTKSourceView* library), *Sublime-Text-3*, and *Visual Studio Code* (*VSCode*)*,* as well as the command-line pager program, *less*. Additionally, *bioSyntax* functions as a repository into which syntax highlighting definition files may be deposited by the community for additional scientific file formats.

### File specifications

At its core, bioSyntax is a set of syntax-highlighting definition files specific to each port which are themselves a programmed set of regular expressions.

Where available, syntax-files are designed using a combination of; official file specifications for SAM v1.5 (https://samtools.github.io/hts-specs/SAMv1.pdf), VCF v4.2 (https://samtools.github.io/hts-specs/VCFv4.2.pdf) PDB v3.30 (ftp://ftp.wwpdb.org/pub/pdb/doc/format_descriptions/Format_v33_Letter.pdf), BED6 (https://genome.ucsc.edu/FAQ/FAQformat.html), and GTF v2.2 (http://mblab.wustl.edu/GTF22.html); example files from databases (NCBI Nucleotide/Protein [26], Sequence Read Archive [27], dbSNP [28], RefSeq [29], RCSB [5] and UCSC Genome Browser [23]); publically available consortium data (1000 genomes project [30], ENCODE [31]); and standard outputs from commonly used software (*samtools* [7], *GATK* [32], *bowtie2* [33], *cufflinks* [34] and *ClustalX* [35]).

### Using bioSyntax

Within each program, highlighting of FASTA, FASTQ, CLUSTAL, BED, GTF, PDB, VCF and SAM files is automatically detected by file extension and can also be manually set within text-editors for files with non-standard file-extensions. In *gedit, sublime* and *vim,* amino acid FASTA files can be coloured using CLUSTAL [35], Taylor [36], Zappo [19] or hydrophobicity [20] colour schemes.

**Fig. 1:**
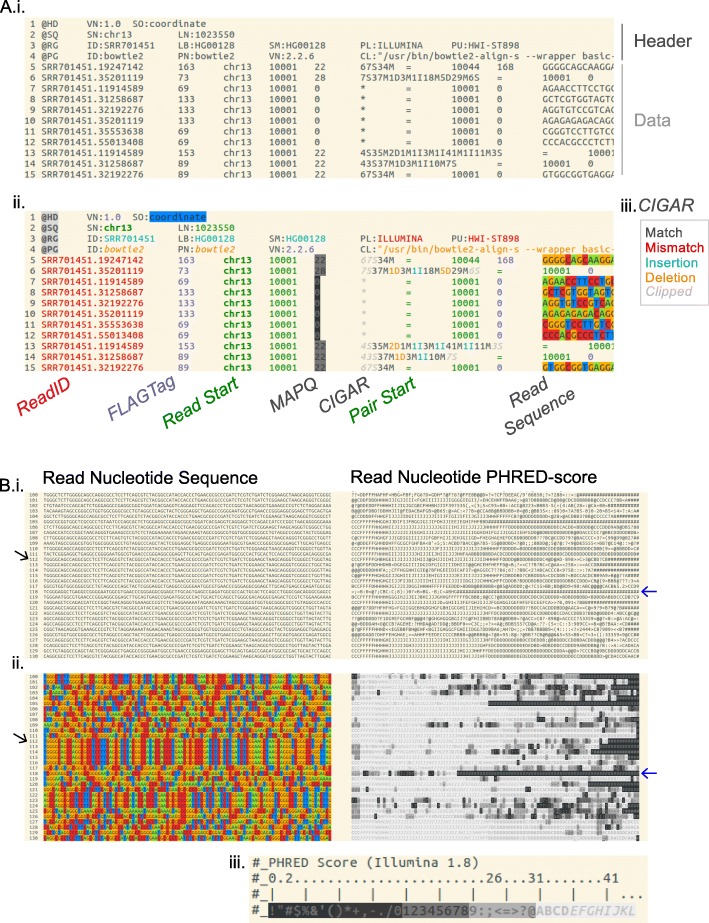
Syntax highlighting for sequence alignment map (.sam) file format. **a** Terminal screenshot of the *‘less HG00128_hgr1.sam’* command run i. normally or ii. with bioSyntax. Related information in the header and data sections are grouped by colours (genomic coordinates, green; sample information, pale blue ...) to improve legibility. Each data-row is an individual sequencing read. Iii. CIGAR alignment strings in particular can be highlighted such that they become substantially easier to read. **b** A broad view of the nucleotide and PHRED-score for 30 reads i. before, and ii. after syntax highlighting. Underlying information of about the data becomes intuitively visible such as PCR-duplicates (black arrow) and poor quality areas and reads (blue arrow) based on iii. PHRED score

Large and compressed data can be manipulated on a unix command pipe with the output directly ported into *less* using explicit bioSyntax format commands: `*sam-less*`, `*vcf-less*`, etc. (Fig. 1). For example `*samtools view -h NA12878.bam | sam-less -* `, or `*gzip -dc gencode.v26.gtf.gz | grep ‘MYC’ - | gtf-less -x 10* `.

## Results and discussion

### Syntax highlighting for computational biology file formats

*bioSyntax* currently recognizes FASTA, FASTQ, CLUSTAL, BED, GTF, PDB, SAM and VCF formats across all four text editors and *less*. Upon installation, *bioSyntax* automatically recognizes file-extensions and seamlessly assigns syntax highlighting to these data files.

The main benefit of syntax highlighting is immediately apparent through its increased legibility (Fig. 1, Additional file 1: Figures S1 and S2), especially in the deconstruction of verbose content such as plain-text CIGAR strings (Fig. 1a). In each file-format data is organized using contrasting colours to accentuate keywords or data fields. Nucleotides and amino acids are highlighted with distinct colours allowing for users to read sequences and interpret patterns in the alignment. Data fields containing scores such as PHRED base quality or mapping scores are gradient coloured.

The overall system of highlighting is also designed to group biological classes, even across file formats (Additional file 2: Table S1). For instance, dark-green is reserved for genomic coordinates in BED, GTF, SAM and VCF, so even if a user is unfamiliar with the SAM format, previous experience associating dark-green in BED or GTF will inform them of the meaning of those fields when presented in a SAM file (Additional file 1: Figure S2).

Ultimately, *bioSyntax* aims to help computational biologists comprehend data using graphical highlighting rather than simple syntactic highlighting. When the data does not have to be read per character or per word, but can be viewed as graphical patterns, underlying information in the data becomes salient, similar to alternative nucleotide representations [37, 38]. This is best seen in complex files such as SAM in which PCR-duplicate reads form block patterns and read density can be approximated by the diagonal similarity of reads at a locus (Fig. 1b). In a user-experience survey of bioinformaticians (Additional file 3: Text 1), 98.6% of users (*N* = 72) selected *bioSyntax* highlighted alignments as being easier to identify nucleotide variants compared to a standard monochrome text. Future research on how syntax highlighting can be refined to optimize for user performance is necessary.

### bioSyntax nucleotide representation

**Fig. 2:**
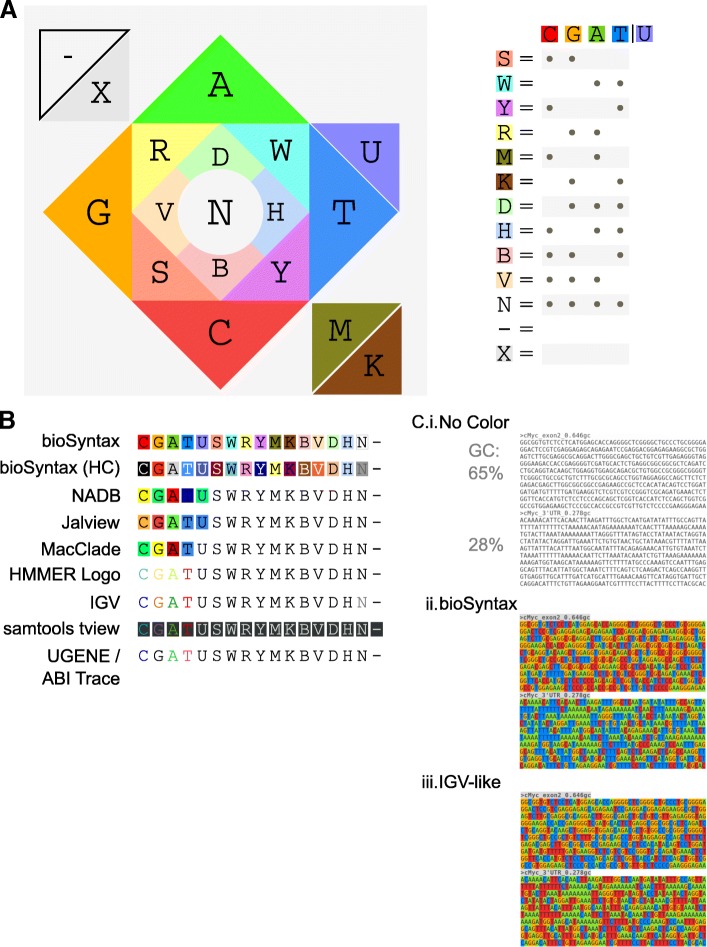
*bioSyntax* nucleotide colour scheme. **a** The four primary bases are coloured in two pairs of contrasting colours. IUPAC ambiguous bases are then coloured in increasingly lighter tones of the approximately mixed colours. To accomodate 4-dimensional bases in 3-dimensional colours, aMino (A or C) and Keto (G or T) bases are darker. **b** A comparison of nucleotide colour-schemes in the literature. **c** bioSyntax colouring allows for approximation of a sequences GC-content by how warm (high GC) or cool (high AT) it appears

*bioSyntax* implements a novel nucleotide colouring scheme for the complete IUPAC ambiguous base set [39], unlike other colour-sets which are designed for four or five bases (Fig. 2). The four primary base colours are chosen such that additive colour mixing also represents complete base ambiguity. For instance, thymine (blue) and cytosine (red) are pyrimidines (magenta), and the “any base”, N, is white. This colour-set visually distinguishes the strong bases (G,C) and weak bases (A,T) as warm and cool colours respectively, allowing for an intuitive approximation of the sequence GC-content (Fig. 2c). Additionally, a high-contrast colour-scheme is available to aid visually impaired or colour-blind users (Additional file 1: Figure S3).

### The *bioSyntax* repository

There are scores of biological and scientific file-formats which would benefit from syntax highlighting. To facilitate future development of syntax definition files in science, the *bioSyntax* repository (https://bioSyntax.org) was set-up. The repository is both a library for scientific syntax highlighting and a community-oriented resource for learning syntax highlighting development. In this manner, researchers experienced in the use-cases of a file-format can quickly develop and share new syntax definition files.

## Conclusions

*bioSyntax* assists researchers to intuitively read and navigate biological files in the context of familiar and common text editing tools. The cross-format unifying colour theme for biological data classes aids users in the rapid and accurate parsing of data with minimal prior knowledge regarding the file-format. Altogether, bioSyntax offers a substantial improvement to the legibility of biological data and helps researchers to grok their data.

## Availability and requirements

- **Project name:** *bioSyntax*
- **Project home page:** https://biosyntax.org
- **Source page:** https://github.com/bioSyntax/bioSyntax
- **Operating system(s):** Linux, MacOS, Windows
- **Programming language:** vim-script, xml, yaml
- **Other requirements:** *gedit* 3.18+ or *GTKSourceView3*-dependent editors; *Sublime-text-3; Visual Studio* Code 1.25+; vim or g*vim* 7.4+; *less* and *source-highlight*
- **License:** GNU General Public License v3.0
- **Any restrictions to use by non-academics:** none

## Additional files

Additional file 1: **Figure S1.** Screenshots of bioSyntax in; A) gedit for the nucleotide sequence FASTA, an amino acid FASTA with CLUSTAL colour scheme and a FASTQ file and; B) sublime-text-3 for a PDB file. **Figure S2.** Screenshots of bioSyntax in; A) vim for the human dbSNP (hg38 build-150) VCF file and; B) less for the Gencode v26 Annotation GTF and C) an example SAM file. In the GTF format, note how background colouring of “start_codon”, “stop_codon”, “CDS”, and “UTR” graphically distinguishes protein-coding transcripts from non-coding transcripts. **Figure S3.** The standard and high-contrast bioSyntax colour set for IUPAC nucleotides under the different forms of simulated colour-blindness. Hue and lightness variations in the standard theme allow for accesibility with colour-blindness. The alternative high-contrast set retains higher visual distinction between bases, even at the monochrome level. (PDF 3125 kb)

Additional file 2: **Table S1.** Biological class definitions and colour definitions for the default bioSyntax theme in hexadecimal (*gedit, sublime, VSCode, and gvim*), cterm (*vim*), and 8-bit ANSI escape character (*less*) colours. (XLS 186 kb)

Additional file 3: **Text 1.** bioSyntax user-experience survey of bioinformaticians. (PDF 3063 kb)

## Abbreviations

GTF: Gene Transfer Format
IUPAC: International Union of Pure and Applied Chemistry
PDB: Protein Database Format
SAM: Sequence Alignment Map
VCF: Variant Call Format
