# Supplementary material for: bioSyntax: syntax highlighting for computational biology
Source: BMC Bioinformatics. 2018 Aug 22;19:303. doi: 10.1186/s12859-018-2315-y (PMC6106740; doi:10.1186/s12859-018-2315-y)

Supplementary Figure 1

A.

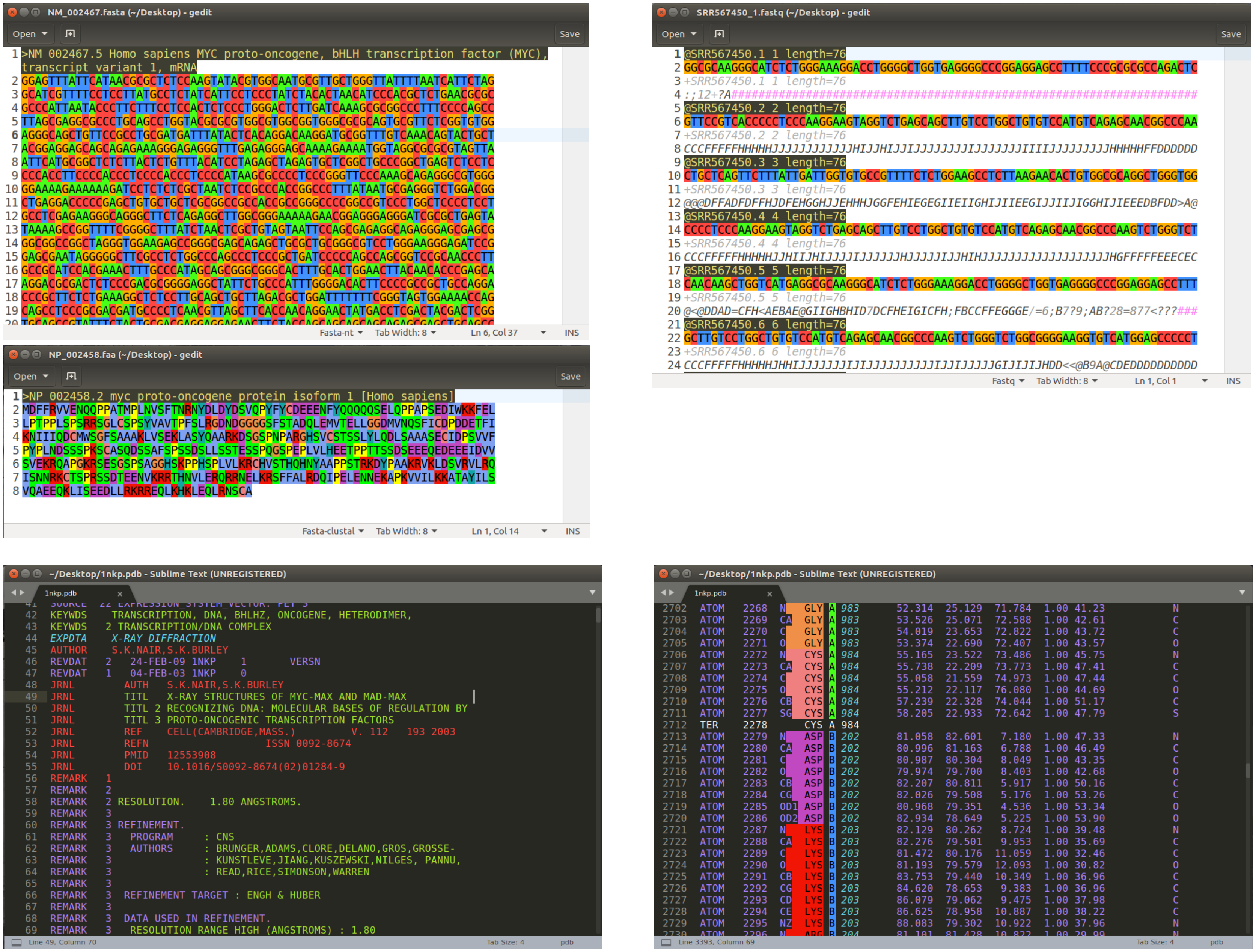

B.

Supplementary Figure 2

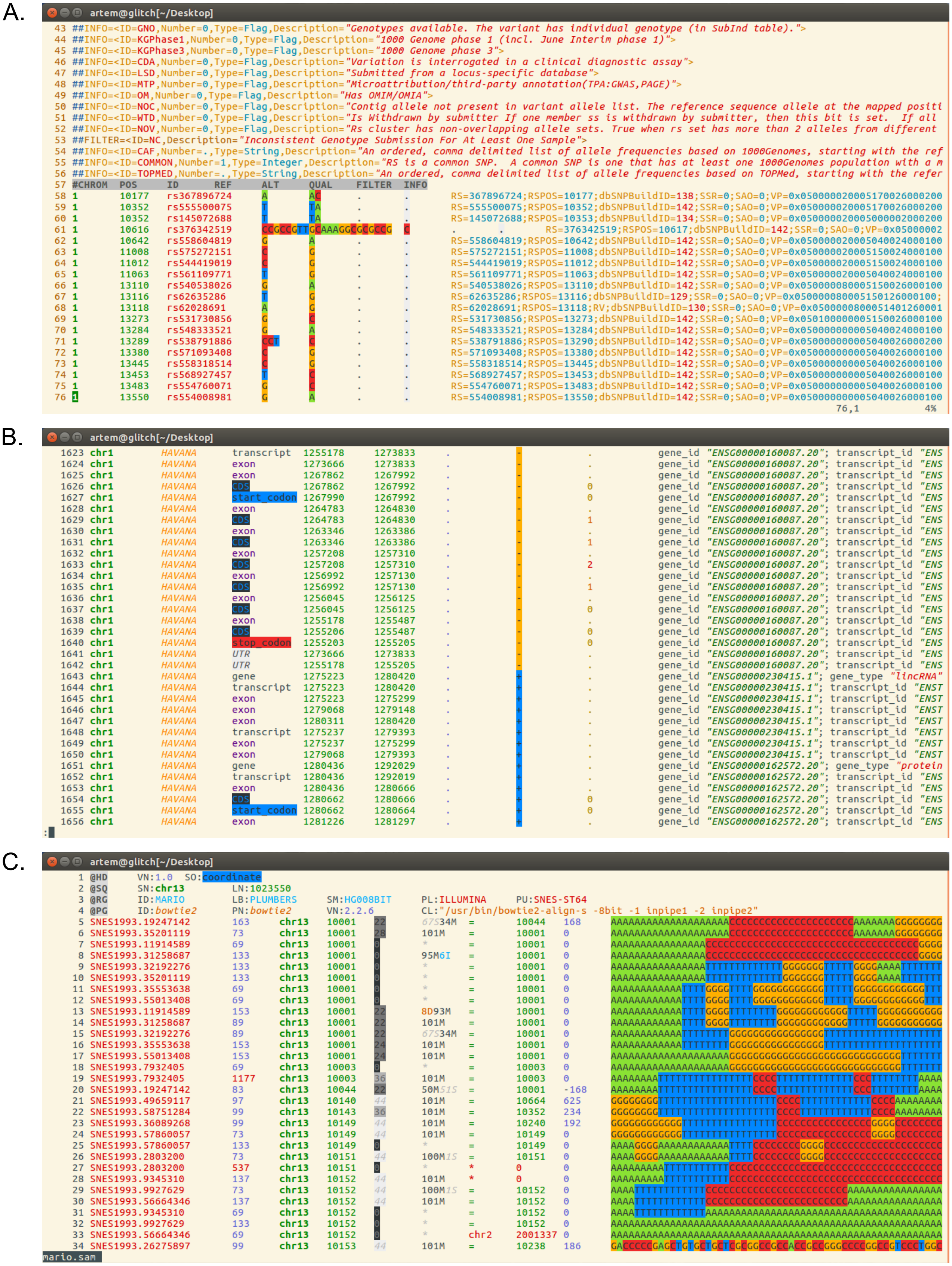

Supplementary Figure 3

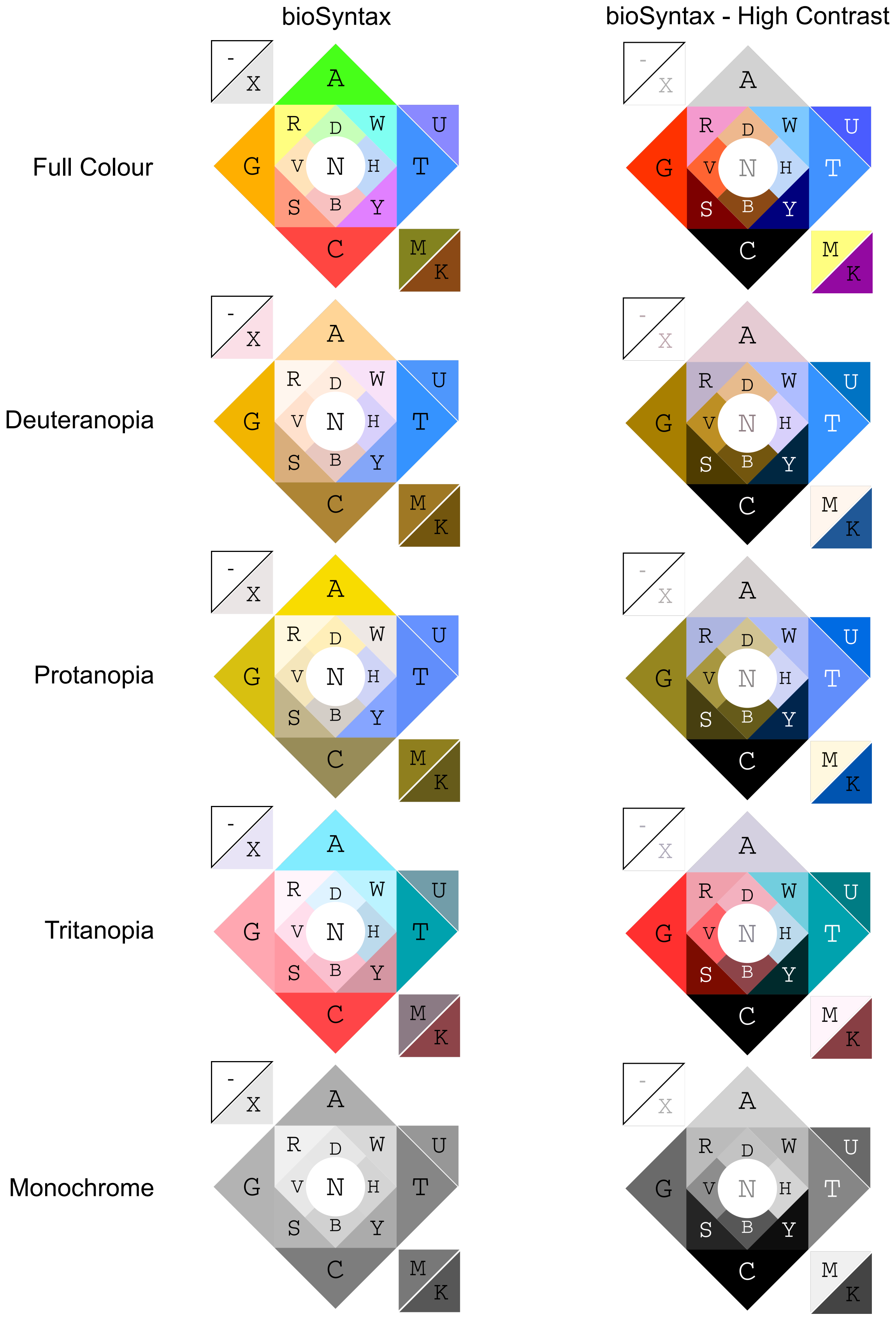

Supplement: Supplementary file 1 — Figure S1. Screenshots of bioSyntax in; A) gedit for the nucleotide sequence FASTA, an amino acid FASTA with CLUSTAL colour scheme and a FASTQ file and; B) sublime-text-3 for a PDB file. Figure S2. Screenshots of bioSyntax in; A) vim for the human dbSNP (hg38 build-150) VCF file and; B) less for the Gencode v26 Annotation GTF and C) an example SAM file. In the GTF format, note how background colouring of “start_codon”, “stop_codon”, “CDS”, and “UTR” graphically distinguishes protein-coding transcripts from non-coding transcripts. Figure S3. The standard and high-contrast bioSyntax colour set for IUPAC nucleotides under the different forms of simulated colour-blindness. Hue and lightness variations in the standard theme allow for accesibility with colour-blindness. The alternative high-contrast set retains higher visual distinction between bases, even at the monochrome level. (PDF 3125 kb) [file 12859_2018_2315_MOESM1_ESM.pdf]
