# Supplementary material for: bioSyntax: syntax highlighting for computational biology
Source: BMC Bioinformatics. 2018 Aug 22;19:303. doi: 10.1186/s12859-018-2315-y (PMC6106740; doi:10.1186/s12859-018-2315-y)
Supplement: Supplementary file 3 — Text 1. bioSyntax user-experience survey of bioinformaticians. (PDF 3063 kb) [file 12859_2018_2315_MOESM3_ESM.pdf]

Figure 1

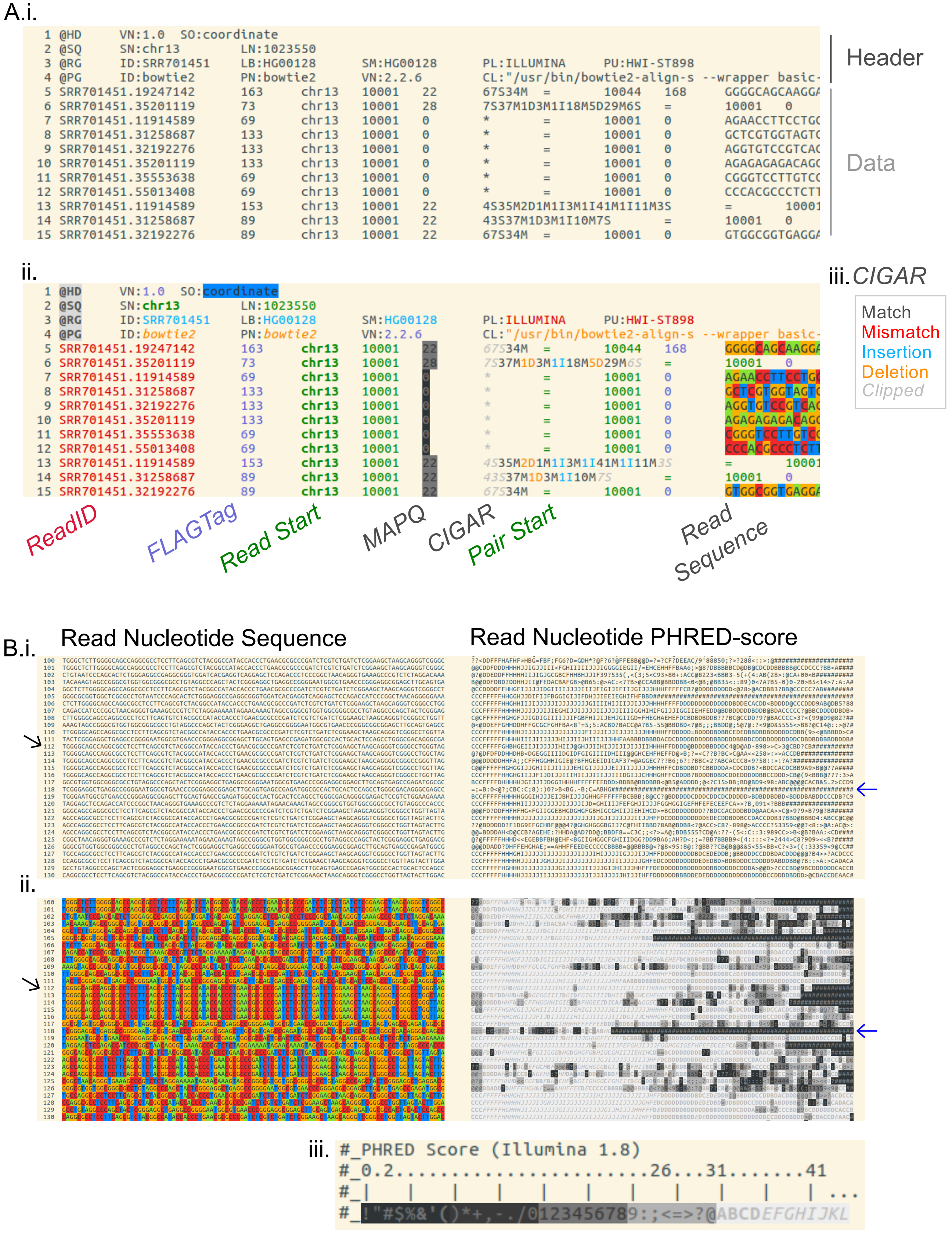
A.i.
1 @HD VN:1.0 SO:coordinate
2 @SQ SN:chr13 LN:1023550
3 @RG ID:SRR701451 LB:HG00128 SM:HG00128 PL:ILLUMINA PU:HWI-ST898
4 @PG ID:bowtie2 PN:bowtie2 VN:2.2.6 CL:"/usr/bin/bowtie2-align-s --wrapper basic-
5 SRR701451.19247142 163 chr13 10001 22 67S34M = 10044 168 GGGGCAGCAAGGA
6 SRR701451.35201119 73 chr13 10001 28 7S37M1D3M1I18M5D29M6S = 10001 0
7 SRR701451.11914589 69 chr13 10001 0 * = 10001 0 AGAACCTTCCTGC
8 SRR701451.31258687 133 chr13 10001 0 * = 10001 0 GCTCGTGGTAGTC
9 SRR701451.32192276 133 chr13 10001 0 * = 10001 0 AGGTGTCCGTCAC
10 SRR701451.35201119 133 chr13 10001 0 * = 10001 0 AGAGAGAGACAGC
11 SRR701451.35553638 69 chr13 10001 0 * = 10001 0 CGGGTCCTTGTCC
12 SRR701451.55013408 69 chr13 10001 0 * = 10001 0 CCCACGCCCTCTT
13 SRR701451.11914589 153 chr13 10001 22 4S35M2D1M1I3M1I41M1I11M3S = 10001
14 SRR701451.31258687 89 chr13 10001 22 43S37M1D3M1I10M7S = 10001 0
15 SRR701451.32192276 89 chr13 10001 22 67S34M = 10001 0 GTGGCGGTGAGGA
Header
Data
ii.
iii. CIGAR
Match
Mismatch
Insertion
Deletion
Clipped
ReadID
FLAGTag
Read Start
MAPQ
CIGAR
Pair Start
Read Sequence
B.i.
Read Nucleotide Sequence
Read Nucleotide PHRED-score
ii.
iii.
#_PHRED Score (Illumina 1.8)
#_0.2.......................26...31.......41
#_| | | | | | | | | | | ...
#_!"#$%&'()*+,-./0123456789:;<=>?@ABCDEFGHIJKL

Figure 2

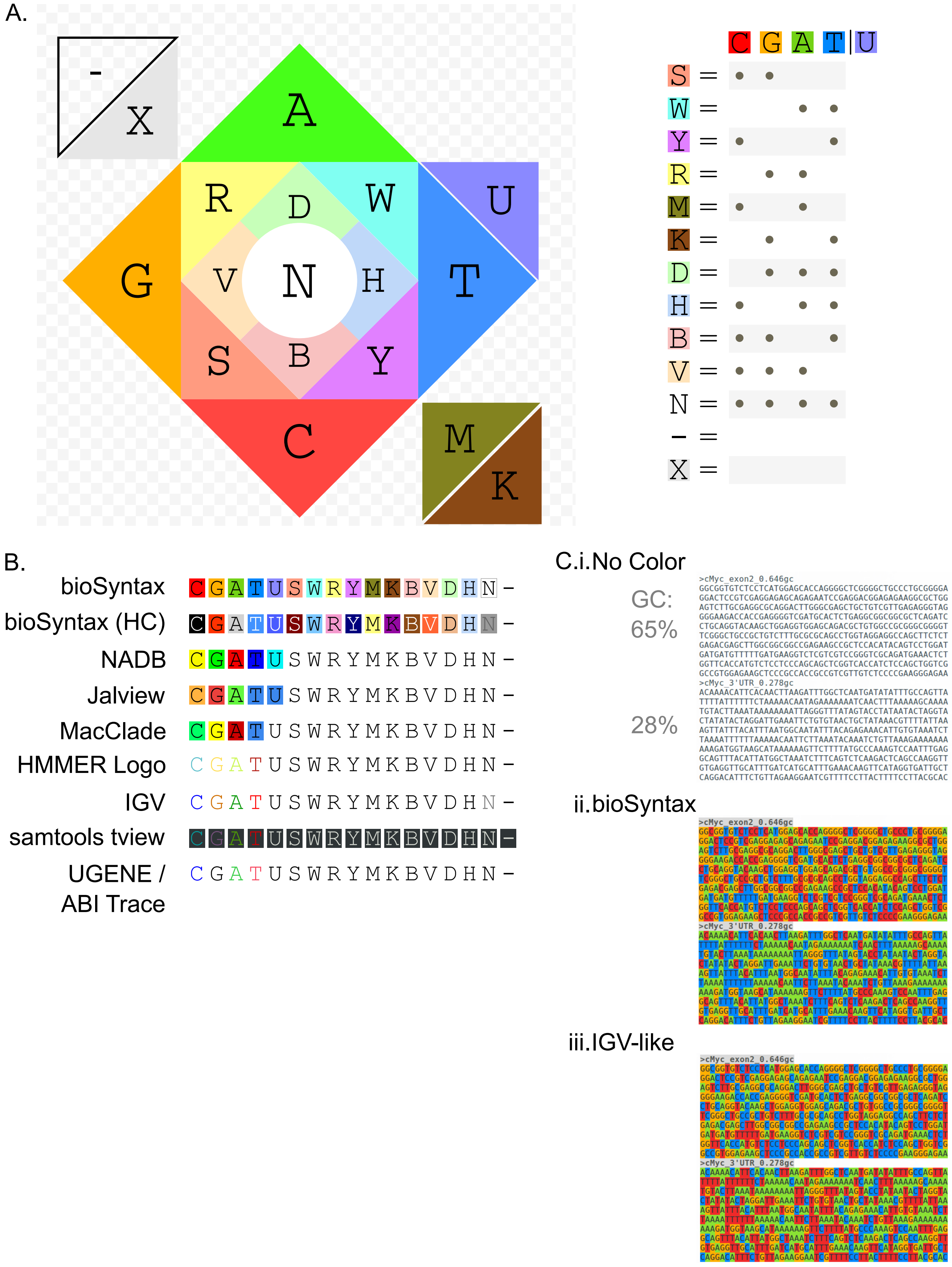
A.
-
X
A
R
D
W
U
G
V
N
H
T
S
B
Y
C
M
K
C G A T U
S =
W =
Y =
R =
M =
K =
D =
H =
B =
V =
N =
- =
X =
B.
bioSyntax
bioSyntax (HC)
NADB
Jalview
MacClade
HMMER Logo
IGV
samtools tview
UGENE / ABI Trace
C G A T U S W R Y M K B V D H N -
C.i.No Color
GC: 65%
28%
ii.bioSyntax
iii.IGV-like
